# Oxygen Sensitivity of Anammox and Coupled N-Cycle Processes in Oxygen Minimum Zones

**DOI:** 10.1371/journal.pone.0029299

**Published:** 2011-12-28

**Authors:** Tim Kalvelage, Marlene M. Jensen, Sergio Contreras, Niels Peter Revsbech, Phyllis Lam, Marcel Günter, Julie LaRoche, Gaute Lavik, Marcel M. M. Kuypers

**Affiliations:** 1 Department of Biogeochemistry, Max Planck Institute for Marine Microbiology, Bremen, Germany; 2 Department of Biological Sciences, University of Aarhus, Aarhus C, Denmark; 3 Department of Marine Biogeochemistry, Leibniz Institute of Marine Sciences (IFM-GEOMAR), Kiel, Germany; Argonne National Laboratory, United States of America

## Abstract

Nutrient measurements indicate that 30–50% of the total nitrogen (N) loss in the ocean occurs in oxygen minimum zones (OMZs). This pelagic N-removal takes place within only ∼0.1% of the ocean volume, hence moderate variations in the extent of OMZs due to global warming may have a large impact on the global N-cycle. We examined the effect of oxygen (O_2_) on anammox, NH_3_ oxidation and NO_3_^−^ reduction in ^15^N-labeling experiments with varying O_2_ concentrations (0–25 µmol L^−1^) in the Namibian and Peruvian OMZs. Our results show that O_2_ is a major controlling factor for anammox activity in OMZ waters. Based on our O_2_ assays we estimate the upper limit for anammox to be ∼20 µmol L^−1^. In contrast, NH_3_ oxidation to NO_2_^−^ and NO_3_^−^ reduction to NO_2_^−^ as the main NH_4_^+^ and NO_2_^−^ sources for anammox were only moderately affected by changing O_2_ concentrations. Intriguingly, aerobic NH_3_ oxidation was active at non-detectable concentrations of O_2_, while anaerobic NO_3_^−^ reduction was fully active up to at least 25 µmol L^−1^ O_2_. Hence, aerobic and anaerobic N-cycle pathways in OMZs can co-occur over a larger range of O_2_ concentrations than previously assumed. The zone where N-loss can occur is primarily controlled by the O_2_-sensitivity of anammox itself, and not by any effects of O_2_ on the tightly coupled pathways of aerobic NH_3_ oxidation and NO_3_^−^ reduction. With anammox bacteria in the marine environment being active at O_2_ levels ∼20 times higher than those known to inhibit their cultured counterparts, the oceanic volume potentially acting as a N-sink increases tenfold. The predicted expansion of OMZs may enlarge this volume even further. Our study provides the first robust estimates of O_2_ sensitivities for processes directly and indirectly connected with N-loss. These are essential to assess the effects of ocean de-oxygenation on oceanic N-cycling.

## Introduction

Oxygen (O_2_) is one of the key regulatory factors of major biogeochemical cycles in the marine environment [1]. The distribution of dissolved O_2_ in the world's oceans is regulated by gas exchange between surface waters and the lower atmosphere, advective processes within the ocean, as well as the biological processes of photosynthesis and respiration. Oxygen, entering the ocean interior mainly at high latitudes, is distributed throughout the global ocean via thermohaline circulation. In the ocean's sunlit surface layer, phytoplankton produces O_2_ and fixes carbon dioxide (CO_2_) in to biomass. Near the base of the euphotic zone, concentrations of O_2_ are generally at their lowest as photosynthesis diminishes or ceases altogether while the repiration of sinking organic matter by heterotrophic micro-organisms consumes O_2_ at maximal rates.

Subsurface regions of severely reduced O_2_ concentrations (O_2_≤5 µmol L^−1^), the so-called oxygen minimum zones (OMZs), are found along the eastern boundaries of the ocean basins in the subtropics and tropics (e.g. off California, Namibia, Peru/Chile) and in the Arabian Sea. Typically in these regions, wind-driven circulation results in the upwelling of nutrient-rich deep waters, fueling high primary production in the euphotic zone. The high surface productivity results in high export of organic matter and thus strong respiration in subsurface waters. Combined with the poor ventilation of these water masses [2], [3], this leads to permanently O_2_-depleted to anoxic conditions at mid-depths [4]–[6].

Although OMZs (if defined by O_2_≤5 µmol L^−1^) account for only ∼0.1% of the global ocean volume [7], they play a key role in controlling the oceans' nutrient inventory as 30–50% of the oceanic nitrogen (N) loss is estimated to occur therein [7], [8]. The recharge of such N-deficient waters from these regions back to adjacent surface waters limits primary production and thus carbon (C) sequestration in large parts of the tropical oceans. N-loss as primarily the formation of gaseous dinitrogen (N_2_) can occur via two pathways: (1) heterotrophic denitrification, the reduction of nitrate (NO_3_^−^) to gaseous dinitrogen (N_2_) via a sequence of intermediates (NO_3_^−^→NO_2_^−^→NO→N_2_O→N_2_) and (2) anammox, the anaerobic oxidation of ammonium (NH_4_^+^) with nitrite (NO_2_^−^) to N_2_. In the OMZs of Namibia and Peru/Chile, on which the current study focuses, anammox has been identified as the major N-loss pathway based on ^15^N-labeling experiments, whereas heterotrophic denitrification was often not detectable or only measured sporadically [9]–[11].

In the course of global climate change and increasing anthropogenic pressures on the marine environment, coastal and open ocean OMZs have been expanding and intensifying in the last decades [12], [13]. A continuing decline in dissolved O_2_ due to reduced O_2_ solubility and enhanced stratification [14], as well as coastal and open ocean eutrophication [15], [16], is expected. De-oxygenation will have the greatest effect on water masses already deficient in O_2_ as these are often at or near the thresholds for anaerobic processes such as anammox or denitrification. Deutsch et al. [17] calculated that a reduction of the mean upper ocean O_2_ content by only 1% would mean a doubling of water masses with O_2_≤5 µmol L^−1^, thus significantly enlarging the ocean volume potentially affected by N-loss.

However, the sensitivities of anammox and denitrification to changes in dissolved O_2_ and their upper O_2_ limits in the marine environment are largely unknown. N-loss attributed to denitrification has been reported to occur at up to 20 µmol L^−1^ of O_2_ [18]. Nonetheless, direct measurements of denitrification under controlled exposure to low O_2_ concentrations in OMZs are lacking. Active anammox bacteria have been found to be abundant at O_2_ concentrations up to 9 and 20 µmol L^−1^ in the Namibian and Peruvian upwelling systems, respectively [9], [10], and it has been suggested that marine snow aggregates could provide suitable anoxic micro-niches at ambient O_2_ concentrations up to 25 µmol L^−1^ [19], [20]. Off Peru/Chile the measured anammox rates were often the highest at the base of the oxycline and in the upper OMZ [10], [11], [21], likely associated with intensified remineralization of organic matter in these water layers. This further indicates that, unlike their cultured counterparts, which are inhibited at O_2_ concentrations as low as 1 µmol L^−1^ [22], marine anammox bacteria can tolerate O_2_ concentrations higher than the upper O_2_ limit (5 µmol L^−1^) often used to restrict anaerobic processes in biogeochemical models [23]. Recently, Jensen et al. [24] investigated the O_2_ sensitivity of anammox in the near-anoxic zone of the Black Sea water column and showed that anammox bacteria remained active up to ∼9 µmol L^−1^ of O_2_. Still unknown is whether this relatively high O_2_ tolerance is widespread amongst anammox bacteria in the major OMZs of the world's oceans.

Although anammox is an autotrophic process, it relies on other N-cycling processes for the required reactive substrates NO_2_^−^ and NH_4_^+^, e.g. NH_3_ oxidation to NO_2_^−^ and heterotrophic nitrate (NO_3_^−^) reduction to NO_2_. The co-occurrence of these aerobic and anaerobic processes together with anammox requires them to be adapted to a certain overlapping range of O_2_ concentrations. Thus far, it remains unclear whether or not processes coupled to anammox can proceed in the same range of O_2_ as assumed for anammox (0–20 µmol L^−1^), or if they show different O_2_ sensitivities that might hence restrict N-loss to a narrower O_2_ regime. Under anoxic conditions, NO_3_^−^ is the next thermodynamically favored electron acceptor, which can be used by a variety of micro-organisms to oxidize organic matter [25]. In OMZ waters, secondary NO_2_^−^ maxima are often interpreted as active NO_3_^−^ reduction [26], [27]. The formation of NO_2_^−^ from NO_3_^−^ is the first step in both denitrification and dissimilatory nitrate reduction to ammonium (DNRA), but it can also be considered as a stand-alone process, as more micro-organisms are known capable of reducing NO_3_^−^ to NO_2_^−^ than to N_2_ or NH_4_^+^ [25], [28]. Heterotrophic NO_3_^−^ reduction to NO_2_^−^ has been measured at high rates in the Peruvian OMZ [29], [30], and has been estimated to account for approximately two thirds of the NO_2_^−^ required for anammox in this region [30]. At the same time, NO_3_^−^ reduction also provides an important source of NH_4_^+^ released from oxidized organic matter [30], [31]. Lipschultz et al. [29] investigated the effect of varying O_2_ concentrations on NO_3_^−^ reduction to NO_2_^−^ in the Peruvian OMZ. They observed that NO_3_^−^ reduction rates doubled under anoxic conditions (N_2_ atmosphere) compared to *in situ* conditions (2.5 µmol L^−1^ of O_2_), while rates decreased by ∼75% at 20 µmol L^−1^ of O_2_.

When O_2_ is present, NO_2_^−^ can be produced aerobically by NH_3_ oxidizing bacteria and archaea in the first step in nitrification. Rates of NH_3_ oxidation are generally highest near the upper OMZ boundaries [32], [33]. In the Peruvian OMZ, this is also where anammox bacteria are most active [10]. These bacteria are partly fueled by NH_3_ oxidation in this zone [30]. A similarly tight coupling between anammox and NH_3_ oxidation was shown earlier for the Black Sea [34]. The occurrence of NH_3_ oxidizers is, however, not restricted to the upper OMZ. They have been found active at non-detectable concentrations of O_2_ (<1–2 µmol L^−1^) in the core of OMZs [30], [33], [35] and are thus obviously well adapted to near-anoxic O_2_ conditions. When Lipschultz et al. [29] investigated the O_2_ sensitivity of NH_3_ oxidation in the Peruvian OMZ, the inferred de-oxygenation of the samples only caused a ∼50% decrease in activity relative to ambient O_2_ (2.5 µmol L^−1^), whereas no stimulation was achieved by an increase to ∼20 µmol L^−1^ of O_2_.

With anammox as well as NO_3_^−^ reduction being apparently tolerant to relatively high O_2_ and NH_3_ oxidation being apparently able to cope with severe O_2_ depletion, an expansion of OMZs might indeed drive larger water masses to greater N-deficits. This would potentially exacerbate N-limitation of primary production in large parts of the ocean and thus affect the oceans' capacity to attenuate the rising atmospheric CO_2_. However, at present no study has systematically investigated the O_2_ sensitivities of anammox and concurrent N-cycling processes in oceanic OMZs, and thus the future nutrient balance in these regions remains speculative at best.

In this paper, we present results for the Namibian and Peru/Chile upwelling systems, two of the most productive regions in the worlds' oceans associated with massive N-loss, where we explored the effect of O_2_ on anammox, NH_3_ oxidation and NO_3_^−^ reduction throughout the OMZ.

## Materials and Methods

### Ethics Statement

The necessary permissions were obtained from the governments of Namibia and Peru to carry out research in their waters.

### Water sampling and nutrient analyses

**Figure 1:**
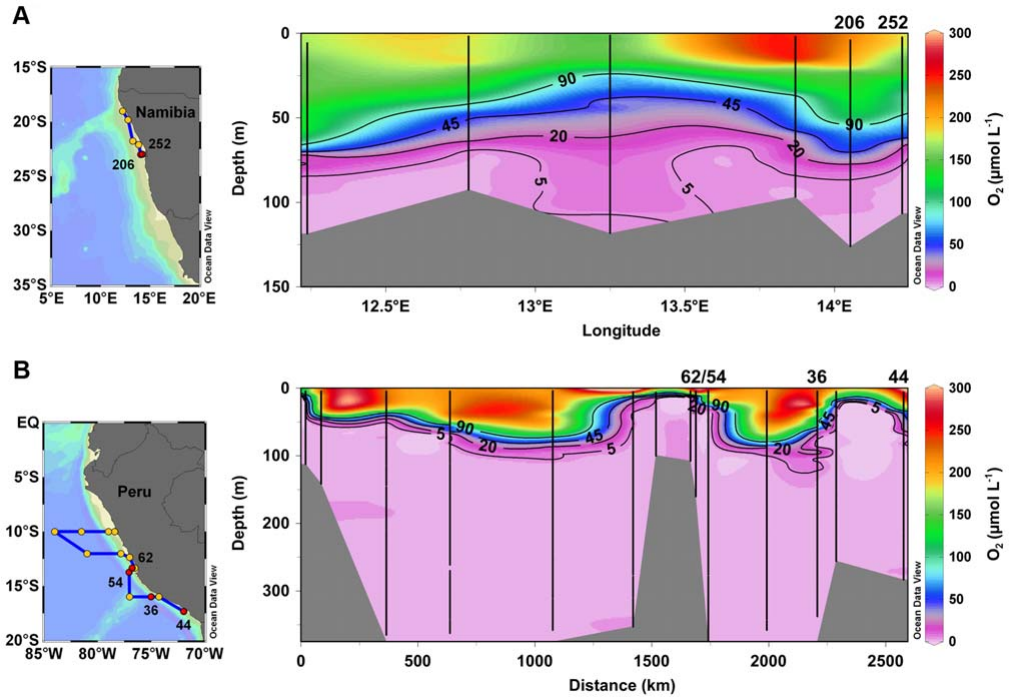
Locations of the sampled stations and distribution of dissolved O_2_. Maps show sampling locations on the A) Namibian shelf and in the B) OMZ off Peru during cruises M76-2 and M77-3, respectively. Water samples were collected by pump-CTD (max. sampling depth: ∼375 m). The oxygen sensitivities of anammox and coupled N-cycling processes were investigated at sampling stations indicated by numbers (red circles). Vertical distributions of dissolved O_2_ are plotted along blue lines.

Samples were taken on two cruises to the OMZs off Namibia (M76/2) and Peru (M77/3), where upwelling persists year-round, onboard R/V Meteor in May/June 2008 and December/January 2008/2009, respectively (Fig. 1). A pump-CTD system was used to collect water samples just below the oxycline, through the core of the OMZ, down to ∼375 m depth off the coast of Peru. The pump CTD system was equipped with a conventional amperometric O_2_ micro-sensor to obtain vertical profiles of dissolved O_2_. In addition, the recently developed STOX (Switchable Trace amount OXygen) sensor [6], which allows high-accuracy O_2_ measurements in near-anoxic environments (detection limit: 50–100 nmol L^−1^ during our deployments), was deployed. At least five measuring cycles after ≥10 min sensor equilibration at a given sampling depth were used to calculate O_2_ concentrations. Water samples were taken with a depth resolution of 1–2 m for nutrient analyses. NH_4_^+^ was measured fluorometrically [36] and NO_2_^−^ was analyzed spectrophotometrically [37] on board. Water samples for NO_3_^−^ and PO_4_^3−^ were stored frozen until spectrophotometric determination [37] with an autoanalyzer (TRAACS 800, Bran & Lubbe) in a shore-based laboratory. Detection limits for NH_4_^+^, NO_2_^−^, NO_3_^−^ and PO_4_^3−^ were 10, 10, 100 and 100 nmol L^−1^, respectively. N-deficits were calculated from the measured fixed inorganic N- and PO_4_^3−^ concentrations as N* (in µmol L^−1^) following Gruber and Sarmiento [8]: N* = [NH_4_^+^]+[NO_2_^−^]+[NO_3_^−^]−16×[PO_4_^3−^]+2.9 µmol kg^−1^×density in kg L^−1^.

### ^15^N labeling experiments

Incubation experiments were carried out at two shallow shelf stations off Namibia (St. 206 and 252) and four stations off Peru (St. 36, 44, 54 and 63), ranging from coastal to open ocean settings (Fig. 1 and Table 1). Based on O_2_ profiles, three to six depths per station were chosen for a standard series of ^15^N-labeling experiments. The experimental procedure for ^15^N-labeling experiments has been described in detail previously [9], [31], [38]. Briefly, N-loss by either anammox or heterotrophic denitrification was measured as the production of ^15^N-labeled N_2_ in ^15^NH_4_^+^ (+^14^NO_2_^−^), ^15^NO_2_^−^ (+^14^NH_4_^+^) and ^15^NO_3_^−^ (+^14^NO_2_^−^) (isotopes: Campro scientific) time-series incubations carried out in 12-ml Exetainers (Labco, UK). At each time interval (about 0, 6, 12, 24 and 48 h) production in one replicate Exetainer was terminated by the addition of saturated mercuric chloride to stop biological activity. The N-isotopic composition of N_2_ gas produced in these experiments was determined by GC/IRMS (Fisons VG Optima). Afterwards, rates of NH_3_ oxidation to NO_2_^−^ and those of NO_3_^−^ reduction to NO_2_^−^ were determined in the same samples as net ^15^NO_2_^−^ production in ^15^NH_4_^+^+^14^NO_2_^−^ and ^15^NO_3_^−^+^14^NO_2_^−^ incubations respectively. The N-isotopic composition of NO_2_^−^ was determined by GC/IRMS after conversion to either nitrous oxide (N_2_O) by sodium azide [39], or to N_2_ by sulfamic acid [40], [41]. Rates were calculated from the slope of linear regression of ^15^N-production as a function of time. Only significant and linear production of ^15^N-species without an initial lag-phase was considered (*t*-tests, *p*<0.05; R^2^>0.8). The net production rates presented here have been corrected for the mole fractions of ^15^N in the original substrate pools but not for isotope dilution due to any other concurrent N-consumption or production processes in the course of the incubation.

**Table 1:** Concentrations of O_2_, NH_4_^+^, NO_2_^−^ and N-conversion rates in ^15^N-labeling experiments in the OMZs off Namibia and Peru.

	Station (water depth)					NH_3_ oxidation†	NO_3_^−^ reduction†	Anammox†	
	[latitude/longitude]	Depth (m)	*in situ* O_2_ § ‡	NH_4_^+^ §	NO_2_^−^ §	^15^NH_4_^+^+^14^NO_2_^−^	^15^NO_3_^−^+^14^NO_2_^−)^	^15^NH_4_^+^+^14^NO_2_^−^	^15^NO_2_^−^+^14^NH_4_^+^
**Namibian**	**M76-206 (131 m)**	90	3.39±0.15	0.01	0.21	29±2*	81±9*	36±1*	13±2*
**OMZ**	**[23.01°S/14.05°E]**	100	2.14±0.10	0.02	0.60	44±1*	103±19*	107±2*	149±5*
		110	0.60±0.11	2.01	0.90	84±5*	97±23*	144±10*	153±4*
	**M76-252 (111 m)**	76	1.11±0.25	0.12	0.14	93±9	370±111	42±15	43±8*
	**[23.00°S/14.23°E]**	95	0.00±0.10	2.24	3.43	110±1	385±21	355±8	399±4*
		105	0.00±0.10	2.51	3.83	92±26	339±77	496±15	462±32*
**Peruvian**	**M77-36 (2845 m)**	90	1.49±0.11	0.05	0.12	35±3	42±2		2.3±0.4
**OMZ**	**[16.00°S/75.00°W]**	120	1.17±0.11	0.05	0.04	1.2±0.1	22±2		19±8
		150	0.60±0.10	0.04	0.02	0.5±0.1	7.2±1.0		0.00
		180	0.00±0.05	0.06	2.96	0.0	39±3		19±3
		250	0.01±0.05	0.06	3.36	0.0	48±13		10±3
		337	0.00±0.05	0.04	0.45	0.0	48±7		0.0
	**M77-44 (281 m)**	75	0.73±0.09	0.14	0.01	19±4	no data	5.1±0.3	
	**[17.34°S/71.94°W]**	87	0.75±0.10	0.09	0.01	21±2	166±15	18±2	
		125	0.02±0.04	0.07	0.28	0.8±0.1	126±8	14±2	
		150	0.01±0.03	0.06	0.30	0.0	87±17	7.4±1.8	
		200	0.02±0.03	0.07	0.33	0.0	19±5	23±2	
		280	0.01±0.04	0.07	5.50	0.0	145±32	7.8±0.6	
	**M77-54 (1893 m)**	41	3.64±0.10	0.06	0.28	47±2	72±3	5.8±1.7	
	**[13.75°S/77.03°W]**	75	0.00±0.05	0.03	0.93	5.0±0.4	71±1	6.3±2.0	
		100	0.00±0.04	0.04	4.01	0.0	71±8	3.0±0.2	
		200	0.00±0.04	0.03	4.87	0.0	0.0	9.4±2.4	
		300	0.00±0.04	0.04	5.75	0.0	0.0	2.6±0.4	
		376	0.00±0.05	0.03	0.46	0.0	77±2	2.2±0.1	
	**M77-62 (160 m)**	40	9.97±0.10	0.40	0.57	0.2±0.1	108±16		25±3
	**[13.35°S/76.75°W]**	50	2.56±0.10	0.08	2.30	15±2	83±2		52±2
		70	0.07±0.04	0.05	1.49	4.6±0.1	89±15		78±4
		100	0.00±0.05	0.04	1.34	2.0±0.2	81±8		39±2
		130	0.00±0.04	0.05	3.45	1.7±0.2	215±6		44±1
		160	0.00±0.05	0.05	4.10	0.0	117±8		108±11

*No addition of ^14^N-species.

§ In µmol L^−1^.

‡ Determined with STOX sensor.

† In nmol N L^−1^ d^−1^.

### Oxygen sensitivity experiments

In order to determine the effect of varying O_2_ concentrations on N-cycle processes, one to two depths per station were sampled for additional O_2_ sensitivity experiments. Samples were taken from the upper OMZ, where aerobic and anaerobic N-cycle processes have been shown to co-occur [30], except one sample taken deeper in the core of the Peruvian OMZ (St. 36). Samples were obtained in 250-mL serum bottles and purged with helium (He) for approximately 15 min to remove any initial O_2_ and to lower the N_2_ background in order to enhance the detection limit of ^29^N_2_ and ^30^N_2_ [38]. As a small sample volume was lost during He-purging, the bottles were then refilled with a second He-purged sample from the same depth to avoid headspace. Afterwards, air-saturated water from the same depth was added to the serum bottles in exchange for part of the de-oxygenated water to adjust samples to the desired O_2_ concentration. At St. 206 and 252 (Namibian OMZ) three samples each were adjusted to ∼3.5, 7.5 and 12 µmol L^−1^ of O_2_, whereas at St. 36, 44, 54 and 63 (Peruvian OMZ) the experimental setup was extended and five samples each were adjusted to ∼1.5, 3, 6, 12, and 24 µmol L^−1^ of O_2_. One sample, to which no air-saturated water was added, served as an anoxic control at all stations. After additions of either ^15^NH_4_^+^+^14^NO_2_^−^, ^15^NO_2_^−^ (+^14^NH_4_^+^) or ^15^NO_3_^−^+^14^NO_2_^−^, samples were transferred into replicate vials (Exetainers, Labco) for time-series incubations. Except for the incubations with only ^15^NO_2_^−^, ^14^N-species were added to all experiments to exclude substrate limitation, which would otherwise complicate the interpretation of any O_2_ effects on the processes of interest. Moreover, keeping the ^14^N-pool of the product of a certain reaction well above the expected concentrations produced from the added ^15^N-substrate could minimize any further conversion of the newly formed ^15^N-products by co-occurring processes. The rate measurements for the various processes were carried out as described above. To exclude formation of ^29^N_2_ due to coupled nitrification-denitrification in incubations amended with ^15^NH_4_^+^ we added allylthiourea (ATU; final concentration 84 µmol L^−1^) to an additional sample of the highest O_2_ treatment (∼11.5 µmol L^−1^) at St. 206 and 252. ATU is a specific inhibitor of aerobic NH_3_ oxidation [42]–[44] and does not affect anammox activity shown at least in sediments [45]. Two sets of incubations were performed in parallel at St. 206 and 252 and one sample per time-point was sacrificed to measure dissolved O_2_. For the remaining stations, O_2_ concentrations were determined only for the initial time-point in each ^15^N-incubation experiment. We used a custom-built, fast-responding O_2_ micro-sensor (Clark-type; MPI Bremen) for most measurements (detection limit: ∼0.5 µmol L^−1^ of O_2_), except at St. 206 where a STOX sensor was used for selected samples.

### Data analysis

We applied least-squares fitting to each set of samples of the O_2_ sensitivity experiments using Excel's solver function [46].

## Results

### Hydrochemistry in the Namibian OMZ

**Figure 2:**
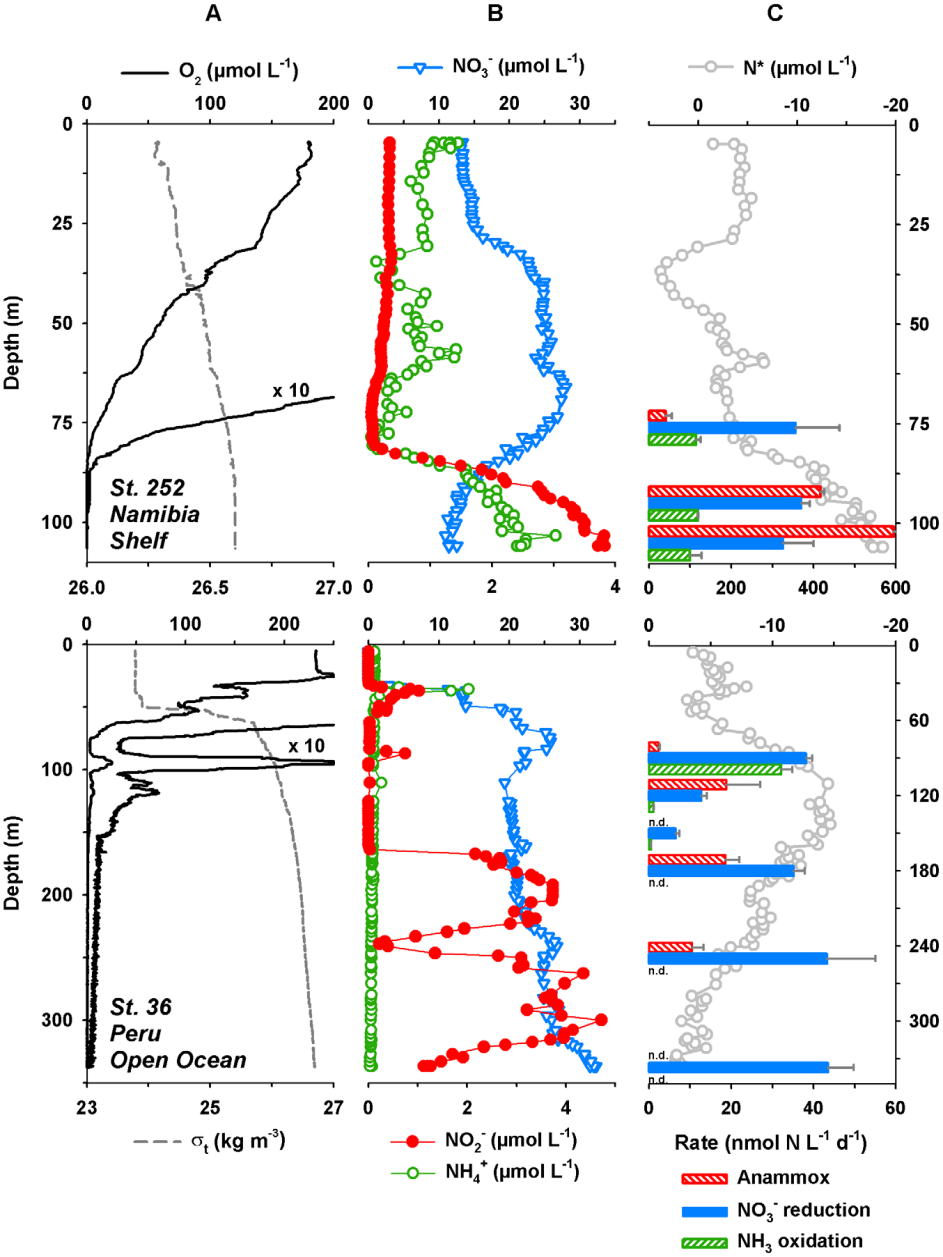
Physicochemical zonation and N-conversion rates at selected stations. Stations are plotted for cruises M76-2 and M77-3 to the OMZs off A) Namibia and B) Peru, respectively. Water depths were 111 m at St. 252 and 2845 m at St. 36. N* was calculated from the fixed inorganic N- and PO_4_^3−^ concentrations (data not shown). Anammox rates were determined in ^15^NH_4_^+^ (St. 206) and ^15^NO_2_^+^+^14^NH_4_^+^ incubations (St. 36). All rates are net rates corrected for the percentage of ^15^N in the pool of the respective N-species. Error bars for rates are standard errors calculated from linear regression.

The water column was poorly stratified over the Namibian shelf at St. 206 and 252 during the time of sampling, as indicated by a weak density gradient, along with the vertical profiles of dissolved O_2_ and inorganic N-species (Fig. 2A). At both stations O_2_ declined gradually with depth, from ∼200 µmol L^−1^ in the surface waters to less than 10 µmol L^−1^ at ∼80 m. STOX measurements at the incubation depths revealed O_2_ concentrations as low as 0.60±0.11 µmol L^−1^ at St. 206. In the central OMZ at St. 252 (Table 1), the sensor was at its detection limit (100 nmol L^−1^ of O_2_ during M76-2). Ammonium concentrations were typically in the range of 1–3 µmol L^−1^ in the oxic zone (<80 m) and decreased to 0.1–0.5 µmol L^−1^ at the base of the oxycline (Fig. 2B). Towards the sediment-water interface NH_4_^+^ concentrations increased up to 4.5 (St. 206) and 2.5 µmol L^−1^ (St. 252). Nitrite concentrations were fairly constant in the upper ∼100 m (0.1–0.5 µmol L^−1^) and increased to ∼2 and ∼4 µmol L^−1^ in the bottom waters at St. 206 and 252, respectively. The increase in both NO_2_^−^ and NH_4_^+^ in the lower OMZ was accompanied by a sharp decrease in NO_3_^−^ concentrations, with minimum concentrations of ∼12 µmol L^−1^ in the lowest sampling depths at both stations.

### Hydrochemistry in the Peruvian OMZ

The stations sampled in the Peruvian OMZ were located on the shelf (St. 62), shelf edge (St. 44) and in the open ocean (St. 36 and 54). Similar to the Namibian shelf stations, the shallowest site (St. 62) was characterized by low density gradients and a gradual decline in O_2_ between ∼20 and 50 m. In contrast, the water column was highly stratified further offshore. Strong pycnoclines, centered around 65, 30 and 55 m at St. 44, 54 and 36, respectively, and a steep oxycline indicated oxygenated surface waters and OMZ were well separated (Figure 2A). Oxygen decreased from ∼250 µmol L^−1^ in the surface to less than 10 µmol L^−1^ at 66 (St. 44), 35 (St. 54) and 75 m (St. 36). A local O_2_ maximum (10 to 25 µmol L^−1^) was found between 90 and 100 m at St. 36, likely due to some lateral advection of more oxygenated water. At all four stations, STOX measurements at the incubation depths revealed traces of O_2_ in the central OMZ at best; mostly here O_2_ concentrations remained below the detection limit of the STOX sensor (∼50 nmol L^−1^ of O_2_ during M77-3). Ammonium concentrations were low and typically 0.05 to 0.1 µmol L^−1^ throughout the OMZ as well as in the surface layer (Fig. 2B). On the shelf, concentrations of NH_4_^+^ were slightly elevated at the base of the oxycline (up to ∼0.4 µmol L^−1^ at St. 62). At the open-ocean stations (St. 54 and 36) NH_4_^+^ maxima of ∼2 µmol L^−1^ were measured at 20 and 35 m, which coincided with NO_2_^−^ maxima (up to 1 µmol L^−1^). In general, NO_2_^−^ concentrations in the surface waters remained below 0.5 µmol L^−1^, whereas NO_2_^−^ accumulated to over 5 µmol L^−1^ in the core of the OMZ at all stations. Nitrate concentrations were as low as ∼1 µmol L^−1^ on the shelf (St. 62). Further off-shore less pronounced NO_3_^−^ concentration minima were detected (∼12 at St. 44 and ∼20 µmol L^−1^ at St. 54 and 36).

### N-cycling in the Namibian and Peruvian OMZs

#### Distribution of anammox activity

Over the Namibian shelf a strong increase in the N-deficit was observed below the oxycline. Minimum values for N* (down to −19 µmol L^−1^) were found in the central OMZ, suggesting N-loss therein. We measured ^15^N^14^N formation in all of our ^15^NH_4_^+^ (+^14^NO_2_^−^) and ^15^NO_2_^−^-incubations at the three depths sampled per station (Table 1). Corrected for the labeling percentage (i.e. the mole fraction of ^15^N in the respective N-substrate pool), rates were comparable in ^15^NH_4_^+^ and ^15^NO_2_^−^ experiments. As no increase in ^15^N^15^N was detectable in either ^15^NO_2_^−^ or ^15^NO_3_^−^ incubations, the formation of ^15^N-labeled N_2_ was attributed to anammox activity and not denitrification. At both stations, anammox rates and N-loss inferred from N* increased with depth (Fig. 2C). Rates ranged from 13 to 43 nmol N L^−1^ d^−1^ at the base of the oxycline to 144 to 496 nmol N L^−1^ d^−1^ in the central OMZ and were generally higher at St. 252.

In the OMZ off Peru, the N-deficit was strongest over the shelf (N* = −33 µmol L^−1^; St. 62) and less pronounced towards the open ocean (N* = 10 µmol L^−1^; St. 54), indicating the highest N-loss likely occurred near the coast. Six depths per station were sampled and ^15^N^14^N formation in ^15^NH_4_^+^+^14^NO_2_^−^ and ^15^NO_2_^−^+^14^NH_4_^+^ was measured in 22 out of 24 incubation depths (Table 1). No formation of ^15^N-labeled N_2_ was detectable at 150 and 337 m at St. 36. As for the Namibian OMZ, whenever N_2_ formation occurred all of the ^15^N-labeled N_2_ produced was recovered as ^29^N_2_ and there was no detectable increase in ^15^N^15^N over time detected in either ^15^NO_2_^−^ or ^15^NO_3_^−^ incubations. Thus, anammox was the only detectable active N_2_-producing pathway, while there was no clear evidence for denitrification activity at the time of our sampling. In general, high anammox activity corresponded with more negative N*, i.e. a more pronounced N-deficit (Fig. 2C). Over the Peruvian shelf, anammox rates (25 to 108 nmol N L^−1^ d^−1^; St. 62) were comparable to those measured over the Namibian shelf (St. 206). Further offshore in the Peruvian OMZ, rates dropped to as low as one tenth of those measured near the coast (2.2 to 9.4 nmol N L^−1^ d^−1^; St. 54).

#### Distribution of nitrate reduction to nitrite activity

Nitrate reduction was measured as ^15^NO_2_^−^ production in all ^15^NO_3_^−^+^14^NO_2_^−^ incubations carried out in the OMZ overlying the Namibian shelf. Nitrate reduction occurred uniformly over the three sampled depths, at rates around 100 and 360 nmol N L^−1^ d^−1^ at St. 206 and 252, respectively (Table 1).

Off Peru, NO_3_^−^ reduction could be detected in 21 out of 23 ^15^NO_3_^−^+^14^NO_2_^−^ incubation experiments. The vertical distribution of NO_3_^−^ reducing activity was slightly variable and high NO_3_^−^ reduction rates did not always coincide with a noticeable accumulation of NO_2_^−^. Similar to anammox activity, maximum rates of NO_3_^−^ reduction were generally detected over the shelf (up to 215 nmol N L^−1^ d^−1^) and decreased towards the open ocean (up to 48 nmol N L^−1^ d^−1^).

#### Distribution of ammonia oxidation activity

Ammonia oxidation, measured as ^15^NO_2_^−^ production in ^15^NH_4_^+^+(^14^NO_2_^−^) incubation experiments, was detected at all incubation depths (Table 1). At St. 206 ^15^N-labeling experiments were carried out under anoxic conditions, whereas samples were incubated at *in situ* O_2_ (<1 µmol L^−1^) at St. 252. Rates increased with depth at St. 206 (from 29 to 84 nmol N L^−1^ d^−1^) but remained rather constant at St. 252 (∼100 nmol N L^−1^ d^−1^).

Off Peru, NH_3_ oxidation to NO_2_^−^ was determined in ^15^NH_4_^+^+^14^NO_2_^−^ incubations under anoxic conditions (St. 44 and 54) or at *in situ* O_2_ levels (St. 36 and 62). Maximum NH_3_ oxidation rates ranged between 15 and 47 nmol N L^−1^ d^−1^. There was no obvious trend in nitrifying activity between coastal and open-ocean stations. Ammonia oxidation was generally confined to the upper OMZ, where O_2_ was still measurable. However, despite an apparent lack of O_2_ *in situ* (i.e. O_2_ concentrations were below detection) shipboard experiments revealed NH_3_ oxidation activity also at St. 54 at 75 m as well as in the central OMZ at St. 62 (1.7 to 5.0 nmol N L^−1^ d^−1^).

### Oxygen sensitivity of anammox and coupled N-cycle processes

#### Oxygen sensitivity of anammox

**Figure 3:**
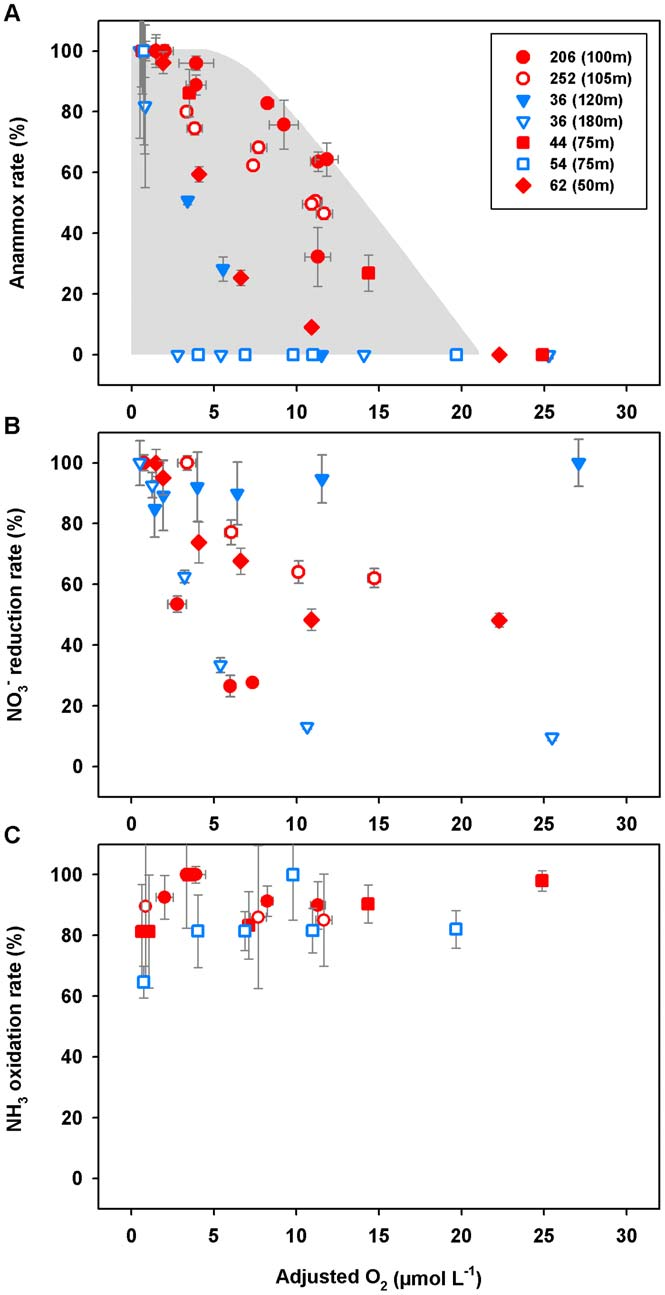
N-cycle processes in Namibian and Peruvian OMZ waters with respect to dissolved oxygen. A) Anammox measured as ^15^N^14^N production in ^15^NO_2_^−^ (+^14^NH_4_^+^) and ^15^NH_4_^+^+^14^NO_2_^−^ incubations. B) NO_3_^−^ reduction measured as ^15^NO_2_^−^ production in ^15^NO_3_^−^+^14^NO_2_^−^ incubations. C) NH_3_ oxidation measured as ^15^NO_2_^−^ production in ^15^NH_4_^+^+^14^NO_2_^−^ incubations. N-conversion rates are given as percentages of the highest rate observed ( = 100%) for the different O_2_ treatments at each incubation depth. Adjusted O_2_ concentrations were verified by micro-sensor measurements. Parentheses in figure legend indicate the corresponding sampling depths at each station shown. Station numbers with double digits and triple digits represent the Peruvian and Namibian stations, respectively. Shelf and open ocean stations are represented by red and blue symbols, respectively. The O_2_ sensitivity assays indicate an upper O_2_ limit for N-loss due to anammox of ∼20 µmol L^−1^ (grey shading).

Anammox activity, as indicated by ^15^N^14^N production from ^15^NH_4_^+^ and ^15^NO_2_^−^, was measurable in all O_2_ manipulation experiments without lag phase at the Namibian shelf stations (Table 2). Oxygen concentration and N_2_ formation showed a significant negative correlation for the incubations with ^15^NH_4_^+^ as well as ^15^NO_2_^−^ at St. 206 and the one with ^15^NH_4_^+^ at St. 252 (Pearson r = −0.95 to −0.99, P<0.05). Similar responses to increased O_2_ were observed for the incubations amended with ^15^NH_4_^+^ and ^15^NO_2_^−^ at both stations. Activity decreased with increasing O_2_ and was, on average, ∼85%, ∼70% and ∼50% of the anoxic control at ∼3.7, ∼8.1 and ∼11.3 µmol L^−1^ of oxygen, respectively (Fig. 3A). Over the course of the incubation (0–48 h) O_2_ concentrations in the ^15^N-labeling experiments did not vary significantly (±0.44 µmol L^−1^ on average). No substantial difference in ^15^N^14^N production was observed between ^15^NH_4_^+^-labeled incubations with and without ATU. This indicates that anammox rather than coupled nitrification-denitrification was the process responsible for the production of ^15^N-labeled N_2_ at 11–12 µmol L^−1^ of dissolved O_2_.

**Table 2:** Rates of NH_3_ oxidation, NO_3_^−^ reduction and anammox measured at varying concentrations of dissolved O_2_.

		NH_3_ oxidation		NO_3_^−^ reduction		Anammox			
Substrate additions:		^15^NH_4_^+^+^14^NO_2_^−^		^15^NO_3_^−^+^14^NO_2_^−^		^15^NH_4_^+^+^14^NO_2_^−^		^15^NO_2_^−^+^14^NH_4_^+^	
		O_2_ § ‡	Rate†	O_2_ § ‡	Rate†	O_2_ § ‡	Rate†	O_2_ § ‡	Rate†
**Namibian**	**M76-206**	2.0	70±5	0.8	65±2	2.0	122±3	0.8	119±10 *
**OMZ**	**(100 m)**	3.9	76±2	2.8	35±2	3.9	108±4	3.9	114±3 *
		8.2	69±4	6.0	17±2	8.2	101±2	9.2	90±10 *
		11.3	68±6	7.3	18±1	11.3	77±4	11.3	38±12 *
	+ATU					11.8	78±7		
	**M76-252**	0.9	92±26	3.4	192±4	0.9	361±12	1.5	430±18 *
	**(105 m)**	3.3	103±18	6.0	148±8	3.3	289±7	3.8	320±9 *
		7.7	89±24	10.1	123±7	7.7	246±7	7.4	267±5 *
		11.7	88±16	14.7	119±6	11.7	167±7	11.1	217±8 *
	+ATU					10.9	179±7		
**Peruvian**	**M77-36**			1.4	22.3±2.5			0.6	10.1±1.2
**OMZ**	**(120 m)**			1.9	23.5±3.1			0.8	8.2±2.7
				4.0	24.2±3.0			3.4	5.1±0.1
				6.4	23.7±2.7			5.5	2.8±0.4
				11.5	24.9±2.1			11.5	0
				27.1	26.3±2.0			25.3	0
	**M77-36**			0.5	38.7±2.9			0.5	15.8±4.5
	**(180 m)**			1.3	35.9±1.6			0.8	12.9±2.5
				3.2	24.2±0.8			2.8	0
				5.4	13.0±0.9			5.4	0
				10.6	5.1±0.3			14.1	0
				25.5	3.8±0.4			25.3	0
	**M77-44**	0.6	12.0±2.3			0.6	4.1±0.6		
	**(75 m)**	1.1	12.0±2.7			1.1	no data		
		3.5	14.7±0.2			3.5	3.5±0.3		
		7.1	12.3±1.6			7.1	no data		
		14.4	13.3±0.9			14.4	1.1±0.2		
		24.9	14.5±0.5			24.9	0		
	**M77-54**	0.8	5.6±0.4			0.8	6.3±2.0		
	**(75 m)**	4.0	6.3±0.9			4.0	0		
		6.9	6.3±0.5			6.9	0		
		9.8	7.8±1.2			9.8	0		
		11.0	6.3±0.6			11.0	0		
		19.7	6.4±0.5			19.7	0		
	**M77-62**			1.5	105±5			1.5	33±1.8
	**(50 m)**			1.9	100±6			1.9	31±1.2
				4.1	77±7			4.1	19±0.8
				6.6	71±4			6.6	8.2±0.8
				10.9	51±4			10.9	2.9±0.5
				22.3	51±2			22.3	0

*No addition of ^14^N-species.

§ In µmol L^−1^.

‡ Adjusted concentrations of O_2_, determined by μ-sensor measurements.

† In nmol N L^−1^ d^−1^.

In the OMZ off Peru, ^15^N^14^N production rates in ^15^NH_4_^+^ and ^15^NO_2_^−^ incubations decreased with increasing O_2_ concentrations in all O_2_ manipulation experiments. However, substantial differences in the O_2_ sensitivity of anammox were found between stations. Over the Peruvian shelf, adjusted O_2_ levels and N_2_ production were linearly and negatively correlated up to 14.4 µmol L^−1^ O_2_ at St. 44 (Pearson r = −0.99, P<0.05) and 10.9 µmol L^−1^ at St. 62 (Pearson r = −0.96, P<0.05). No rates were detectable beyond ∼20 µmol L^−1^ of O_2_. At the open-ocean stations in the Peruvian OMZ, anammox activity appeared to be more sensitive to the added O_2_ (Fig. 3A). At St. 36, ∼30% activity of the anoxic control experiment remained detectable when O_2_ was increased from the in situ ∼1.2 µmol L^−1^ (measured by STOX) to 5.5 µmol L^−1^ of O_2_ in the 120 m sample. In comparison, anammox was fully inhibited at 2.8 µmol L^−1^ of O_2_ already in the 180 m sample, where O_2_ was not detectable by the STOX sensor *in situ*. A similarly strong O_2_ response was seen at St. 54, where rates dropped to zero at 4.0 µmol L^−1^ of O_2_ in the 75 m incubation experiment.

#### Oxygen sensitivity of nitrate reduction to nitrite

Nitrate reduction rates in the O_2_ sensitivity assay carried out for the Namibian OMZ waters, decreased with increasing O_2_ concentrations (Table 2). The incubation experiments at St. 206 revealed a stronger negative response to elevated O_2_ levels than those performed at St. 252. Activity at St. 206 was reduced to ∼30% of the anoxic control in the highest O_2_ treatment (7.3 µmol L^−1^), whereas a doubling of the O_2_ concentration (14.7 µmol L^−1^) led to a decrease in NO_3_^−^ reduction rates to ∼60% of the control experiment at St. 252 (Fig. 3B).

In the Peruvian OMZ, production of ^15^NO_2_^−^ from ^15^NO_3_^−^ was never fully inhibited by O_2_, not even in the highest O_2_ treatments (∼25 µmol L^−1^ of O_2_). Nevertheless, NO_3_^−^ reduction rates showed marked differences in their sensitivity towards elevated O_2_ levels between and within our experimental stations. For example at St. 36, NO_3_^−^ reduction activity in the upper OMZ sample (120 m) at St. 36 did not vary significantly among the various O_2_ treatments (1.4 to 27.1 µmol L^−1^ of O_2_), while activity decreased to ∼10% of the control experiment in samples taken deeper (180 m) in the OMZ when adjusted to 25.5 µmol L^−1^ of O_2_ (Figure 3B).

#### Oxygen sensitivity of ammonia oxidation

Rates of NH_3_ oxidation to NO_2_^−^ showed no significant difference over the range of the applied O_2_ concentrations (∼1–12 µmol L^−1^) in the Namibian OMZ samples (Table 2). Activity varied by a maximum of ∼15% among the different O_2_ treatments but without any systematic trends (Fig. 3C).

Similar to the observations for the Namibian shelf, ^15^NO_2_^−^ production in the ^15^NH_4_^+^ experiments conducted for the Peruvian shelf (St. 44) and at open-ocean (St. 54) stations showed no marked differences among the different O_2_ treatments (∼1–25 µmol L^−1^). Only the control experiment (0.8 µmol L^−1^ O_2_) at St. 54 suggested a slightly lower NH_3_ oxidation rate (−35%) compared to the higher O_2_ treatments (Fig. 3C).

## Discussion

### Oxygen sensitivity of anammox in OMZ waters

In the investigated samples from both the Namibian and Peruvian OMZ, the only N_2_-forming pathway detected by ^15^N-labeling experiments was anammox. This confirms the results from earlier studies, which detected N-loss due to anammox but not denitrification in these regions [9]–[11]. The highest anammox rates (on the order of 500 nmol N L^−1^ d^−1^) were measured in the Namibian shelf waters. Off Peru, rates declined from ∼50 nmol N L^−1^ d^−1^ over the shelf to <10 nmol N L^−1^ d^−1^ at the open ocean sites. This may be explained by differences in surface productivity between the two upwelling systems [47] as well as between Peruvian coastal and open-ocean waters, since organic matter transport ultimately fuels all processes delivering NH_4_^+^ and NO_2_^−^ for the anammox reaction [30], [31]. Anammox often showed the highest rates in the upper OMZ, as seen in previous studies [10], [11], [21] probably in response to the high NH_4_^+^ release from the enhanced remineralization of particulate organic matter at the base of the oxycline, below which all three activities decreased with depth. There were exceptions, however, particularly at depths close to the seafloor on the shelf, where exceptionally high rates were likely supported by NH_4_^+^ diffusing out of the sediment [9], [48], [49] (S. Sommer, pers. comm.).

In the O_2_ tolerance assays, N-loss due to anammox was in fact detectable at O_2_ levels significantly higher (up to ∼15 µmol L^−1^) than that generally used to define OMZs (<5 µmol L^−1^ of O_2_). Anammox activity in samples taken at the shallow sites appeared the least affected by increasing O_2_. The rates therein remained measurable even at adjusted O_2_ concentrations of 10 to 15 µmol L^−1^. These are almost twice as high as the anammox O_2_-tolerance level previously determined in the Black Sea suboxic zone [24]. In comparison, anammox activity appeared increasingly sensitive to O_2_ towards the open ocean and deeper in the OMZ, where rates were not detectable above 2.8 to 5.5 µmol L^−1^ of O_2_ (St. 36 and 54). Based on the observed negative linear correlation between the measured rates and adjusted O_2_ levels, the upper O_2_ limit for anammox to proceed in the OMZs is estimated to be ∼20 µmol L^−1^ (Table 3 & Fig. 3).

**Table 3:** Overview of the response of NH_3_ oxidation, NO_3_^−^ reduction and anammox to changes in dissolved O_2_.

Process	Region	Station	Sampled depth (m)	Substrate addition	Upper OMZ boundary (m) †	*in situ* O_2_ §	O_2_ at 50% rate reduction §
**NH_3_ oxidation**	Namibian OMZ	206	100	^15^NH_4_^+^+^14^NO_2_^−^	77	2.1	no trend observed
	Namibian OMZ	252	105	^15^NH_4_^+^+^14^NO_2_^−^	64	0.0	no trend observed
	Peruvian OMZ	44	75	^15^NH_4_^+^+^14^NO_2_^−^	52	0.7	no trend observed
	Peruvian OMZ	54	75	^15^NH_4_^+^+^14^NO_2_^−^	26	0.0	no trend observed
**NO_3_^−^ reduction**	Peruvian OMZ	36	120	^15^NO_3_^−^+^14^NO_2_^−^	51	1.2	no trend observed
	Namibian OMZ	252	105	^15^NO_3_^−^+^14^NO_2_^−^	64	0.0	17.3
	Peruvian OMZ	62	50	^15^NO_3_^−^+^14^NO_2_^−^	26	2.6	14.7
	Peruvian OMZ	36	180	^15^NO_3_^−^+^14^NO_2_^−^	51	0.0	4.1
	Namibian OMZ	206	100	^15^NO_3_^−^+^14^NO_2_^−^	77	2.1	3.6
**Anammox**	Namibian OMZ	206	100	^15^NH_4_^+^+^14^NO_2_^−^	77	2.1	16.0
	Namibian OMZ	252	105	^15^NH_4_^+^+^14^NO_2_^−^	64	0.0	11.0
	Namibian OMZ	206	100	^15^NO_2_^−^	77	0.0	10.9
	Namibian OMZ	252	105	^15^NO_2_^−^	64	2.1	10.6
	Peruvian OMZ	44	75	^15^NH_4_^+^+^14^NO_2_^−^	52	0.7	10.1
	Black Sea*	1	100	^15^NH_4_^+^+^14^NO_2_^−^	∼75	<1	8.6
	Black Sea*	1	100	^15^NO_2_^−^	∼75	<1	7.1
	Peruvian OMZ	62	50	^15^NO_2_^−^+^14^NH_4_^+^	26	2.6	5.8
	Peruvian OMZ	36	120	^15^NO_2_^−^+^14^NH_4_^+^	51	1.2	4.7
	Peruvian OMZ	54	75	^15^NH_4_^+^+^14^NO_2_^−^	26	0.0	2.4
	Peruvian OMZ	36	180	^15^NO_2_^−^+^14^NH_4_^+^	51	0.0	1.9

† Here defined as water depth where O_2_ drops below 25 µmol L^−1^.

§ In µmol L^−1^. Calculated from regression functions obtained by least-squares fitting of the data given in Table 2.

*Jensen et al. 2008.

The apparently higher O_2_ tolerance at the shelf stations may be explained by an adaptation of anammox bacteria to fluctuations in dissolved O_2_ due to the presence of a less stable oxycline at the upper boundary of the OMZ. Vertical mixing is usually enhanced in coastal upwelling regions. This was indicated by a weak density gradients and a gradual O_2_ decline over the Namibian shelf, where the level of dissolved O_2_ are known to be variable [50]. In the open-ocean off Peru, ventilation of the OMZ from above is hindered due to strong stratification [51]. The dissolved O_2_ content is perhaps most stable within the core of the OMZ, where the highest O_2_ sensitivity of anammox was measured in our current study (180 m at St. 36). With O_2_ concentrations consistently below 1–2 µmol L^−1^, anammox bacteria thriving therein are unlikely to have adapted to higher O_2_ levels compared to their counterparts in more dynamic environments.

Alternatively, marine snow particles have been speculated to provide “anoxic” micro-environments in which O_2_ is sufficiently depleted to favor N-loss at ambient O_2_ levels <25 µmol L^−1^ [9], [20], while some anammox bacteria have been shown to be potentially particle-associated in the Namibian OMZ [20]. Hence, higher abundance of particles in coastal waters than further offshore or in the core of the OMZ might also explain the apparently higher O_2_ tolerance by anammox bacteria near the coast.

### Oxygen sensitivity of nitrate reduction in OMZ waters

The reduction of NO_3_^−^ to NO_2_^−^, was detected at high rates at the shallow shelf stations both off Namibia and Peru (∼100 to 360 nmol L^−1^ d^−1^) and decreased with increasing distance from the coast in the Peruvian OMZ (∼10 to 50 nmol L^−1^ d^−1^ at St. 36). The rates measured off Peru are consistent with earlier results from ^15^N-labeling experiments in the same region [29], [30] and a similar rate distribution was recently reported for the Arabian Sea OMZ [52], [53].

Reduction of NO_3_^−^ to NO_2_^−^ showed a high degree of variability in O_2_ sensitivity amongst stations. No effect of increasing O_2_ on NO_3_^−^ reduction was observed in the 120 m incubations at St. 36. At the remaining stations, the correlation between activity and adjusted O_2_ concentrations was non-linear and could be best described by an exponential function, as determined by least-squares fitting (Table 3 & Fig. 3b). Our results from two shelf stations in the Namibian (St. 252) and Peruvian (St. 62) OMZs further confirmed earlier observations by Lipschultz et al. [29] that NO_3_^−^ reduction was only moderately affected by increasing O_2_. About 50% of NO_3_^−^ reduction activity remained when O_2_ was adjusted to ∼14 to 17 µmol L^−1^ in our above-mentioned samples (Table 3). More pronounced sensitivity to O_2_ was detected at St. 206 on the Namibian shelf and at 180 m at St. 36 off Peru, where rates were reduced by ∼50% relative to the control already at ∼4 µmol L^−1^ of O_2_.

The observation, that in general NO_3_^−^ reduction activity was only moderately affected by increasing concentrations of O_2_ may at first seem at odds with the fact that NO_3_^−^ respiration is generally considered an anaerobic process. However, it has been reported from experiments with cultures and environmental samples that complete or partial denitrification can take place under aerobic conditions [54]–[56]. Moreover, the different enzymes involved in the step-wise reduction on NO_3_^−^ to N_2_ during denitrification, differ in their O_2_ sensitivity. In various bacterial strains the NO_2_^−^ and nitrous oxide (N_2_O) reductase appear to be most sensitive with respect to O_2_, whereas the NO_3_^−^ reductase is the most O_2_-tolerant enzyme [57]–[59]. This O_2_ tolerance could explain the observation that even the highest O_2_ additions did not lead to a full inhibition of NO_3_^−^ reduction in the samples taken from the Namibian and Peruvian OMZ waters. However, the detected variability in terms of O_2_ sensitivity among the different incubation experiments and the lack of any response at 120 m at St. 36 remains puzzling. One possible explanation might be the high phylogenetic diversity and thus variable physiology of the NO_3_^−^ reducers inhabiting the OMZ waters [30], [60].

### Oxygen sensitivity of ammonia oxidation in OMZ waters

Ammonia oxidizing activity seemed widespread throughout the OMZ overlying the Namibian shelf, as indicated by high NO_2_^−^ production rates. Off Peru, nitrifying activity peaked at the base of the oxycline, where the highest NH_4_^+^ release due to remineralization of sinking organic matter can be expected. Though O_2_ was not always detectable *in situ*, NH_3_ oxidation rates could be detected at these upper OMZ depths, consistent with previous studies [30], [33], [35].

In the O_2_ sensitivity assays, NH_3_ oxidation at most decreased slightly in the anoxic control (St. 54) when compared to the higher O_2_ treatments. No stimulation at higher O_2_ levels (20 to 25 µmol L^−1^ of O_2_) was achieved. A similar observation was made by Lipschultz et al. [29], though they detected a 50% reduction of activity in their assumedly anoxic control. Our results suggest a relatively high O_2_ affinity of aerobic NH_3_ oxidizers in both OMZs investigated. It has been shown that cultured bacterial NH_3_ oxidizers, including marine nitrifiers, are, in principle, able to cope with very low O_2_ concentrations down to at least ∼2 µmol L^−1^ [61]–[63]. The only cultured marine aerobic ammonia oxidizing archaea investigated so far appears to have a limited capacity to survive under near anoxic conditions [64]. However, a higher O_2_ affinity of archaeal NH_3_ oxidizers in the environment is indicated by results from the Peruvian OMZ, which suggest that both bacterial and archaeal NH_3_ oxidizers are active at undetectable *in situ* O_2_ levels (<1.5–2 µmol L^−1^) [30].

Based on our findings, the minimum O_2_ concentration for NH_3_ oxidizer to be active in OMZ waters is most likely in the nanomolar range. An adaptation of aerobic micro-organisms to extremely low O_2_ has been shown in a recent study by Stolper et al. [65]. They demonstrated aerobic growth in a culture experiment at an O_2_ concentration ≤3 nmol L^−1^. Alternatively, when O_2_ is scarce, NH_3_ oxidizer may also grow anaerobically via the oxidation of NH_3_ with gaseous nitrogen dioxide (NO_2_) or tetraoxide (N_2_O_4_) [66]. However, as these compounds are rare in the marine environment, it is unlikely that this is of major ecological significance.

### Implications for N-loss in the future ocean and our understanding of N-cycling in modern OMZs

In summary, the current study shows that O_2_ is a major controlling factor for anammox activity in OMZ waters. Based on our O_2_ assays we estimate the upper limit for anammox to be ∼20 µmol L^−1^ O_2_, which is significantly higher than previously shown for the Black Sea (Table 3 & Fig. 3). In contrast, NH_3_ oxidation and NO_3_^−^ reduction as the main NH_4_^+^ and NO_2_^−^ sources for anammox were little or only moderately affected by changing concentrations of dissolved O_2_. Intriguingly, aerobic NH_3_ oxidation was active at non-detectable O_2_ concentrations, while NO_3_^−^ reduction to NO_2_^−^, which is generally considered to be an anaerobic process, was fully active up to at least 25 µmol L^−1^ O_2_. Hence, aerobic and anaerobic N-cycle pathways in OMZs can co-occur over a larger range of O_2_ concentrations than previously assumed. The zone where N-loss can occur is primarily controlled by the O_2_-senstivity of anammox and not by the O_2_-senstivity of the tightly coupled aerobic NH_3_ oxidation and anaerobic NO_3_^−^ reduction.

Additionally, our results indicate that N-loss and other N-cycling processes within such O_2_ regimes would be controlled by other environmental factors such as substrate availability. For instance, the (near) anoxic conditions in the core of the OMZ do not confer the highest NO_3_^−^ reduction and anammox rates despite the ideal O_2_ regime. Surface water productivity and therewith export of particulate organic matter into the OMZ might play an important role in controlling anammox activity. Sinking organic matter is the ultimate source of the required reactive substrates NO_2_^−^ and NH_4_^+^ for anammox and it may also provide suitable anoxic micro-environments for anammox bacteria in zones of higher ambient O_2_ [9], [20].

The fact that anammox in the marine environment can proceed at O_2_ levels ∼20 times higher than those known to inhibit enrichment cultures of anammox bacteria (∼1 µmol L^−1^) [22] enlarges the global oceanic volume potentially affected by N-loss from the previously estimated 0.1% tenfold to ∼1% (O_2_≤20 µmol L^−1^) [67]. In addition, recent reports show that OMZs have been expanding and intensifying worldwide, particularly in the tropical Atlantic and Pacific [13]. Such expansions of the OMZs would mean an even greater increase in ocean volume potentially subject to active N-loss processes in the coming years. In other words, progressively more fixed inorganic N may be removed from the oceans, and larger areas in the subtropics and tropics might experience enhanced N-limitation due to the recharge of N-deficient waters back to the surface in the future. In the long run, negative feedbacks might also ensue from increasing N-loss and ocean warming. Less productive surface waters would export less organic matter to subsurface waters and lead to reduced O_2_ consumption rates. The stronger stratification due to the warming of the upper ocean might also hamper upwelling of nutrient-rich water to the surface, therewith reducing export production and the respiration of O_2_ in OMZs.

The relative significance of these positive and negative feedback mechanisms, or how they may counteract each other and eventually influence global oceanic nutrient budgets, would require further investigations complemented with realistic global biogeochemical modeling. To date, the models used to develop future scenarios of the global ocean nutrient balance have rarely taken into account coupling N-cycling processes, and certainly not their respective O_2_ sensitivities.

In light of the above presented results, the simple switching from aerobic to anaerobic respiration at ∼5 µmol L^−1^ of O_2_ often implemented in models [23] appears not realistic. The current study provides the first robust estimates of O_2_ sensitivities for processes directly and indirectly connected with N-loss. These factors are necessary for biogeochemical models to collectively and accurately assess the effects of ocean de-oxygenation on N-cycling in OMZs and neighboring water masses, and hence global oceanic N-balance.
